# Prognostic Value of Stem Cell Quantification in Stage II Colon Cancer

**DOI:** 10.1371/journal.pone.0088480

**Published:** 2014-02-21

**Authors:** Maria Angeles Vaz, Juan Carlos Martinez, José Manuel Devesa, Javier Die Trill, Victor Abraira, Alejandro Riquelme, Alfredo Carrato

**Affiliations:** 1 Medical Oncology Department, Ramon y Cajal University Hospital, Madrid, Spain; 2 Department Pathology, Instituto oftálmico/Hospital Universitario G. Marañón, Madrid, Spain; 3 Department of Surgery, Ramon y Cajal University Hospital, Madrid, Spain; 4 Department of Statistics, Ramon y Cajal University Hospital, Madrid, Spain; Southern Illinois University School of Medicine, United States of America

## Abstract

**Background:**

Cancer stem cells (CSCs) are a subset of tumor cells with capacity to self-renew and generate the diverse cells that make up the tumor. The aim of this study is to evaluate the prognostic value of CSCs in a highly homogeneous population of stage II colon cancer.

**Methods:**

One hundred stage II colon cancer patients treated by the same surgical team between 1977 and 2005 were retrospectively analyzed. None of the patients received adjuvant chemotherapy. Inmunohistochemistry expression of CD133, NANOG and CK20 was scored, using four levels: <10%, 11–25%, 26–50% and >50% positivity. Kaplan-Meier analysis and log rank test were used to compare survival.

**Results:**

The average patient age was 68 years (patients were between 45–92 years of age) and median follow up was 5.8 years. There was recurrent disease in 17 (17%); CD133 expression (defined by >10% positivity) was shown in 60% of the tumors, in 95% for NANOG and 78% for CK20. No correlation was found among expression levels of CD133, NANOG or CK20 and relapse-free survival (RFS) or overall survival (OS). However, a statistical significant correlation was found between established pathological prognostic factors and RFS and OS.

**Conclusions:**

Stem Cell quantification defined by CD133 and NANOG expression has no correlation with RFS or OS in this cohort of Stage II colon cancer.

## Introduction

Colon cancer is one of the most common causes of cancer death worldwide and survival is affected by local recurrence as well as development of metastatic disease.

The cancer stem cell theory is based in the assumption of the existence of a small population of cancer-initiating cells, which is exclusively responsible for the growth and maintenance of the entire tumor. These cells have low proliferative rate, high self-renewal capacity and propensity to differentiate into active proliferating tumor cells and they are resistant to chemotherapy and radiation. These cells have been reported in different cancers.

Recent research findings suggest that the cancer stem cell model also applies for colon carcinoma. Ricci-Vitiani and O’Brien reported that the CD133 positive cells in colorectal cancer exhibit the properties of cancer initiating cells with self-renewal and high tumorogenic potential. These CSCs express pluripotency markers such as CD133 and NANOG and do not express markers of differentiation as cytokeratin20 (CK20) [1], [2], [3].

The potential prognostic value of CSCs in colorectal cancer has been studied with conflicting results. However some of these series were based on heterogeneous situations, involving rectal and colon cancer and different tumor stages (I–IV). The aim of this study is to evaluate the prognostic value of CSCs in a highly homogeneous population of stage II colon cancer.

## Materials and Methods

A total of 100 pathologically confirmed specimens were obtained from colon cancer patients with TNM stage II, who were treated surgically between 1977 and 2005 at the Ramon y Cajal University Hospital. To reduce effects of adjuvant treatment on prognosis none of the included patients received adjuvant chemotherapy. All patients were followed up for recurrence-free survival and overall survival. The follow-up period was calculated from the date of surgery until 2010. Recurrence was defined as re-appearance of the initial tumor; and both metastasis or local recurrence were considered to be evidence of tumor relapse. Overall survival was defined as the time from surgery to death or at the point when the patient was last recorded to be living. In this study known prognostic clinical factors were also registered. High risk patients were considered to be those with at least one of the following features: fewer than 12 lymph nodes present in the surgical specimen, T4 tumor, lymphovascular invasion and poorly differentiated tumors. The TNM classification was used for pathologic staging and the World Health Organization classification was used for pathologic grading. The research has ethics committee approval (Ramon y Cajal University hospital). Written informed consent was obtained for use of the sample in research.

### Histological Examination

Specimens were retrieved from the pathology files of the Ramon y Cajal University Hospital, Madrid, Spain, and histologically reviewed. Paraffin blocks were retrieved to construct tissue arrays containing 1,5 mm diameter tissue cylinders from colon cancer and control specimens. Each tissue array paraffin block contained at least 2 samples of each case in order to have a cross control of signal expression. The most representative areas of each tumor were selected for the study. Reactive lymphoid tissue (tonsils) were used as negative controls of expression and incorporated by triplicate in each tissue array. Histopathological features were independently reviewed. This was followed by common observation on a multihead microscope, where discrepancies were resolved.

### Immunohistochemistry (IHC)

Anti-CD133 Rabbit Monoclonal Antibody (Cell Signaling Technology) and goat polyclonal ant human NANOG (Novus Biologicals), were used to identify stem cell markers. Anti-Cytokeratin20 (CK 20) Mouse Monoclonal Antibody (Dako, Denmark), was used to evaluate differentiated epithelial cells. Immunohistochemical staining was performed following the Dako Real En Vision rabbit/mouse method (Dako, Denmark). As a positive control of pattern expression and signal specificity, we used fetal human brain tissue sections including the brain ventricular neuro epithelium. This was the first place where CD133 expression was detected in the apical border of neuroephitelial cells [4]. Later on, release as membrane particles CD133 positive, was observed in the ventricular fluid and different human body fluids, including gut [5]. Incubation with an unrelated antibody was employed as a control of antigenic preservation and recovery. Incubation omitting the first antibody was used as a negative control of a non-specific detection system staining.

### Immunohistochemical Examination

Immunohistochemistry examination was done from paraffin embedded tissue sections that included the most representative tumor areas. CD133, NANOG and CK20 were analyzed. The tumors were given a semi-quantitative score using four levels: <10%, 11–25%, 26–50% and >50% positivity. We defined a sample to be positive if there were >10% positive cells.

### Statistical Analysis

The Kaplan-Meier analysis was used to estimate cancer-specific survival and the groups were compared with the log-rank test. Statistical procedures were performed using SPSS version 15.0, and p<0.05 was considerer statistically significant. All analyses were performed using the Statistical Package for Social Sciences (SPSS) Software.

## Results

### Clinicopathological Findings

The clinicopathological findings are listed on Table 1. The median age of the patients was 68 years old (patients range from 45 to 92 years), 53% were males and 47% were females.

**Table 1:** Clinical and pathological data of the stage II colon cancer patient cohort (n = 100).

Characteristics		N of Patients %
Age		68 years (45–92)
Gender	Male	53%
	Female	47%
CD133	<10%	40%
	11–25%	22%
	26–50%	16%
	>50%	22%
NANOG	<10%	5%
	11–25%	9%
	26–50%	34%
	>50%	51%
High risk 52%	<12 Lymph nodes	35%
	T4/T3	93%/4%
	Poorly differenciated histology	G1 74%/G2 12%/G3 9%
	Lymphovascular invasion	11%
Recurrence	Yes	17%
	No	83%
Death	Yes	39%
	No	61%
Cause of death	No death	62%
	Colorectal cancer	16%
	Other causes	20%

Known high risk clinicopathological findings for recurrence were registered, defined by fewer than 12 lymph nodes present in the surgical specimen, T4 tumors, lymphovascular invasion and poorly differentiated tumors, and patients were categorized in the high risk category if there were one or more of these risk factors or in the low risk category if there were no risk factors. Fifty two percent were high risk and 48% were low risk patients. Thirty five percent of patients had <12 lymph nodes in the surgical specimen. Only 4% were T4, 9% of patient had poorly differentiated histology and 11% had lymphovascular invasion. At the time of last follow-up 17% of patients had recurrent disease and 61% were alive; 36% were dead, 16% of colorectal cancer and 20% of other causes.

### The CD133 Antigen Location in Colon Cancer

**Figure 1:**
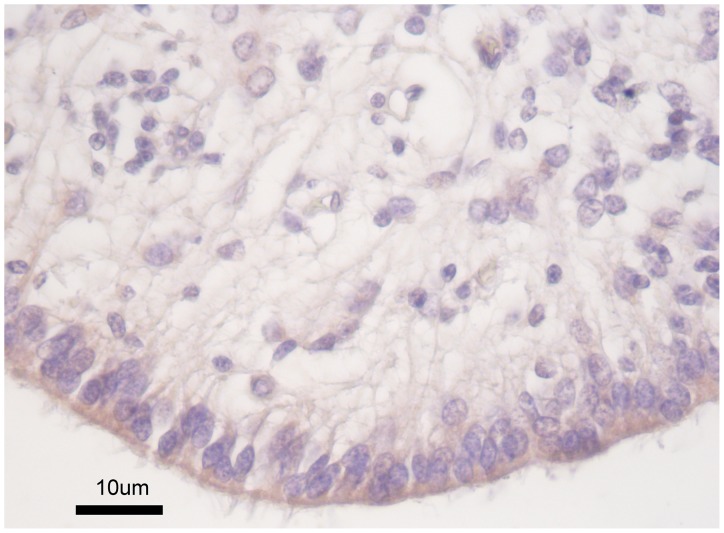
CD 133 protein expression in the apical border of periventricular neuroepithelial cells (Positive control).

The positive inmunohistochemical reactivity pattern for CD133 observed in periventricular neuroepithelial cells (control cases) (Fig. 1), is in keep to that observed in previous studies [5], and acts as a positive control of the specificity of the signal. The CD133 antigen has previously been shown to be located in apical plasma membrane protusions of the cultures cancer cell line Caco-2 [5].

We observed a polar staining of CD133 confined to the apical luminal cell surface of colorectal cancer glands. Usually several CD133 positive cells were grouped together. CD133 expression tended to be pronounced close to the invasive margin of the tumors.

**Figure 2:**
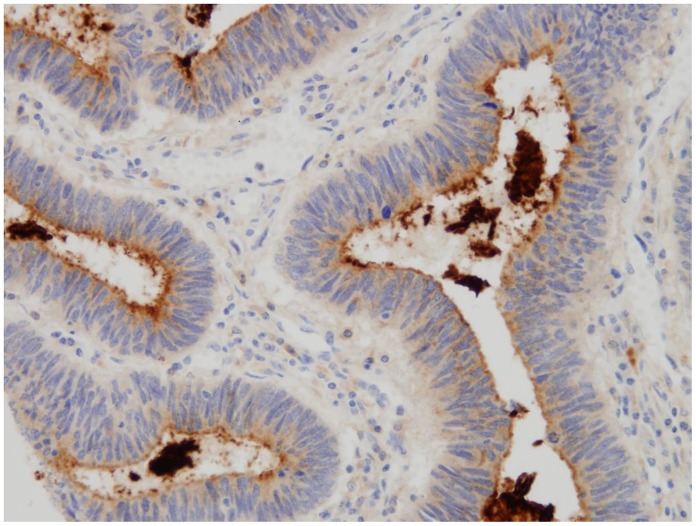
CD 133 protein expression in the apical border of cancer cells, shedding CD 133 positive debris into the luminal glands.

**Figure 3:**
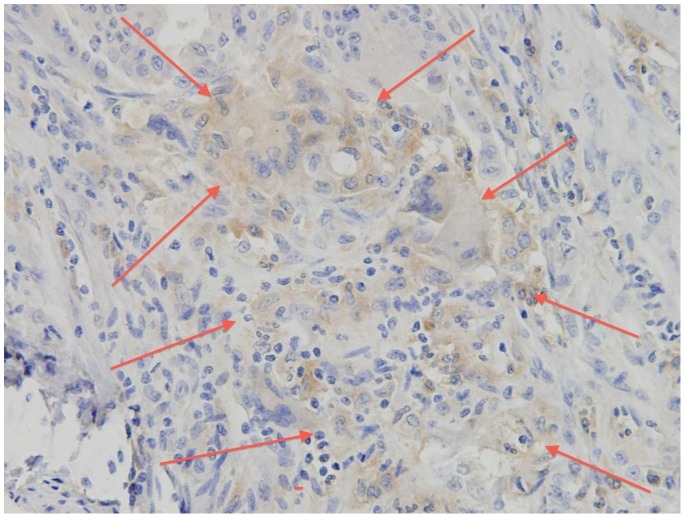
Group of neoplastic cells showing CD 133 positive cytoplasmic expression.

CD133 protein expression was observed in the apical border of colon cancer cells and glands, frequently shedding CD133 positive debris into the luminal glands (Fig. 2). Occasional groups of neoplastic cells showed positive whole cytoplasmic expression (Fig. 3).

No CD133 positive signal was found in any of the tissue tonsil specimens, nor in the mucosal surface of them, used as negative control. This rule out the possibility of false positivity, or non-specific binding of the CD133 antibody used, to mucosal/luminal spaces.

**Figure 4:**
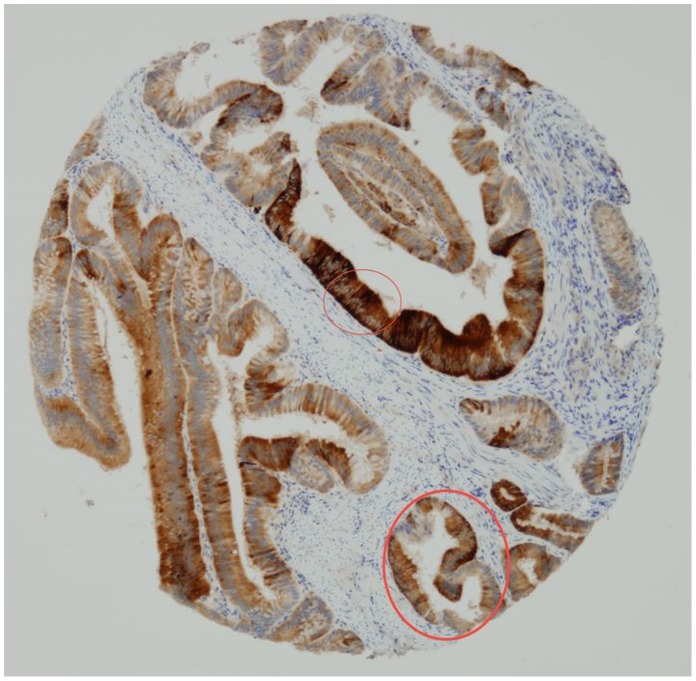
Most neoplastic cells express CD133.

**Figure 5:**
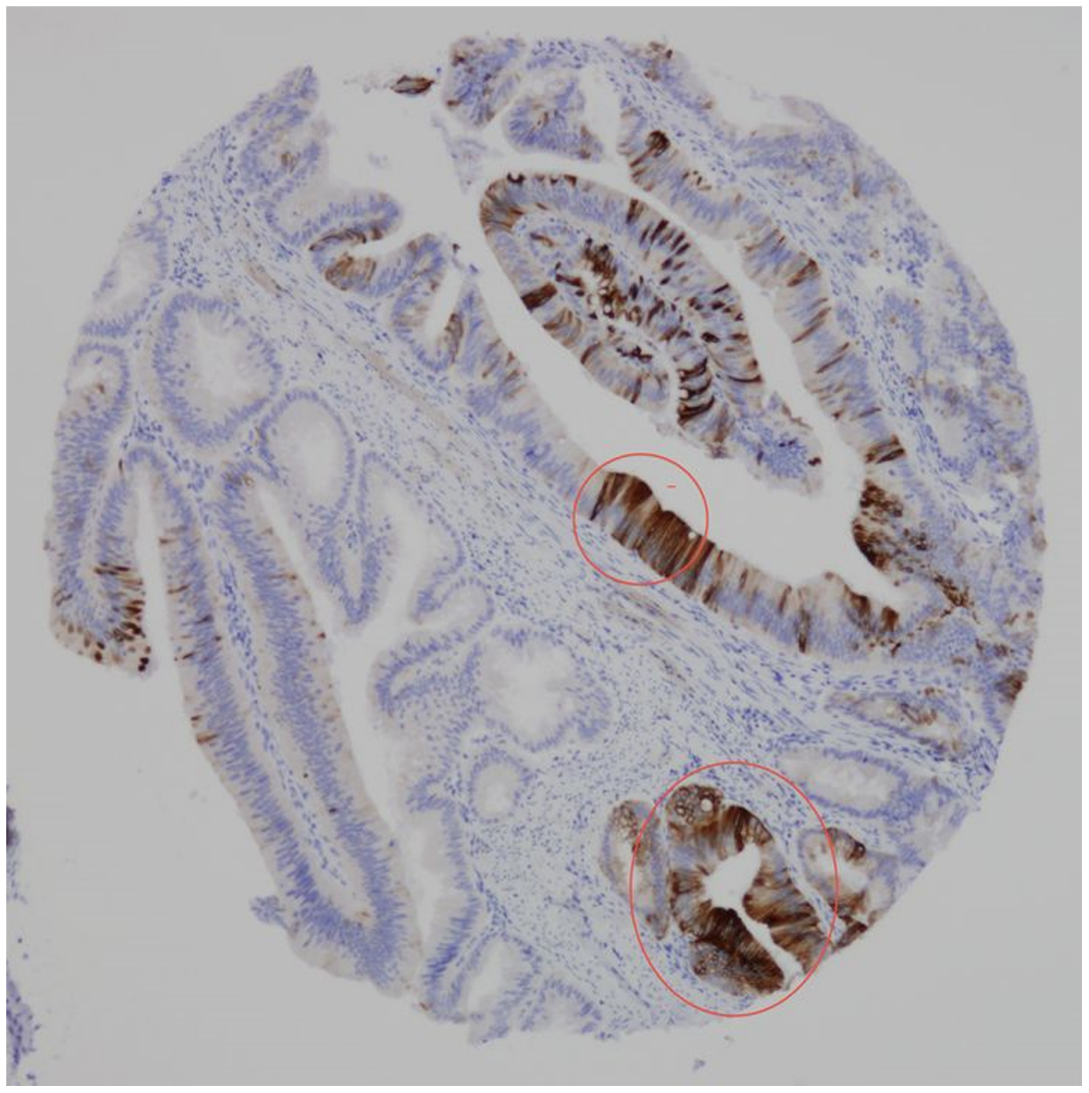
Most neoplastic cells express null or weak CK20. However, there are groups of cells expressing simultaneously CD 133 and CK 20 (encircled areas).

By looking to consecutive tissue sections, we compared CD133 and CK20 protein expression (Fig. 4). Fields containing CD133 positive cells usually showed weak or no CK20 protein expression, while areas with high CK20 expression were usually CD133 negative. However groups of neoplastic cells expressing simultaneously CD133 and CK20 were shown as well (Fig. 5).

### Immunohistochemical CD133 Expression

CD133 expression was detected in 100% of patients. Forty per cent of patients had<10% positivity; 22% of patients had 11–25% positivity; 6% of patients had 26–50% positivity and 22% of patients had >50% positivity.

All 100 patients were followed up for survival to analyze CD133 expression as a prognostic factor. The median follow-up period was 5.8 years. Of the 100 patients, 16.3% died of their cancer and 20,4% of other causes. There were 7 cases of local recurrence, 11 cases of distant metastasis.

**Figure 6:**
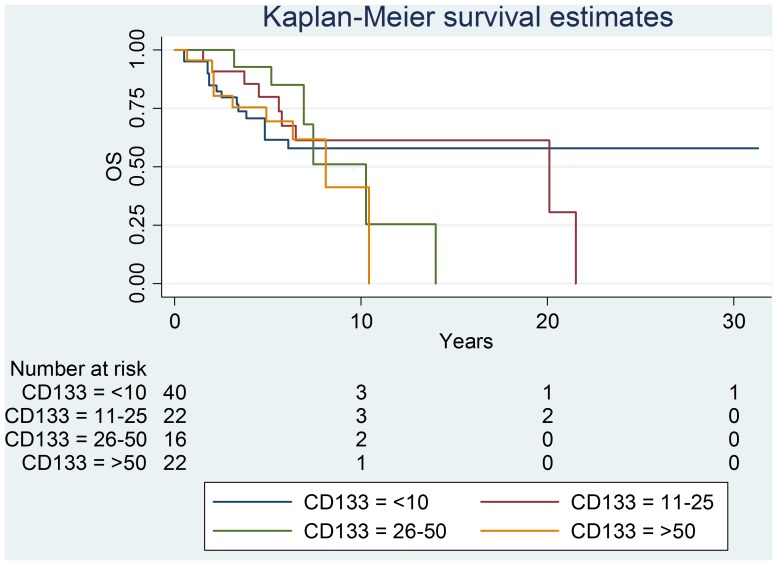
Recurrence-free survival curves and CD133- positivity expression.

Recurrence-free survival curves were not different between the different levels of CD133 positivity expression. Kaplan-Meier curve for overall survival showed no statistical differences between the different groups (fig. 6).

### NANOG Expression

NANOG nuclear and cytoplasmic expression was detected in 99% of patients. Five patients had<10% positivity; 9 patients had 11–25% positivity; 34 patients had 26–50% positivity and 51 patients had >50% positivity. Positive staining was mainly observed at he epithelial neoplastic cells, many of them coincidental with CD133 positive cells. However, NANOG positive/CD133 negative neoplastic cells were shown as well. Moreover, NANOG expression was also observed in neoplastic stroma cells (data not shown).

**Figure 7:**
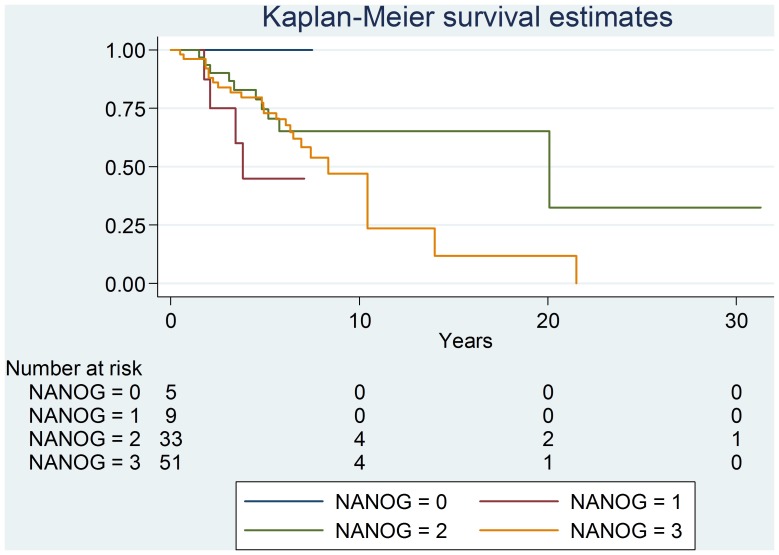
Kaplan-Meier curve for recurrence and survival.

Kaplan-Meier curve for recurrence and overall survival showed no statistical differences between the different levels of expression (Fig. 7).

### CK20 Expression

CK20 expression was detected in 78% of patients. Twenty-two per cent of patients had<10% positivity; 29% of patients had 11–25% positivity; 26% of patients had 26–50% positivity and 23% of patients had >50% positivity.

Kaplan-Meier curve for recurrence and survival showed no statistical differences between the different groups of expression.

**Figure 8:**
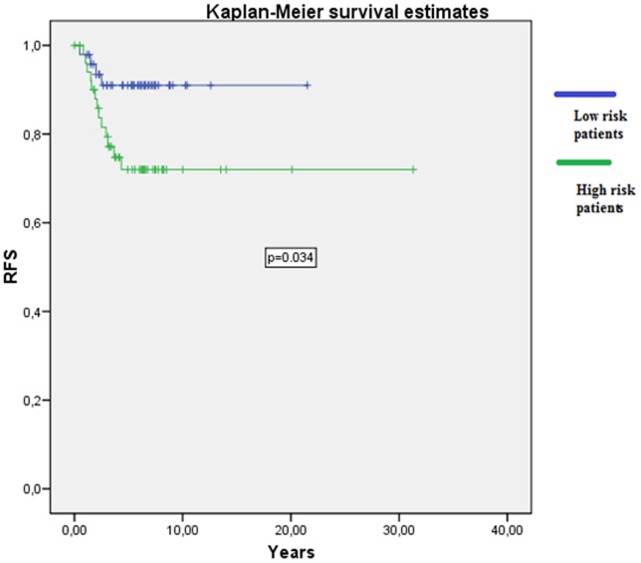
Correlation between known clinical factors and RFS.

The relationship between high risk clinicopathological known factors for patients, defined as having at least one of the following: fewer than 12 lymph nodes present in the surgical specimen, T4 tumors, lymphovascular invasion and poorly differentiated tumors, and recurrence and survival were analyzed. A statistical significant correlation was found between known clinical factors and RFS and OS (p = 0.034) (Fig. 8).

## Discussion

CD133 is the epitope of a glycosilated form of human prominin-1, which is a member of the pentaspan transmembrane glycoprotein [1], and expression of CD133 has been reported to be localized in normal neonatal and adult precursor cell as well as cancer initiating cells [2], [3]. Remarkably, prominin-1 is specifically associated with plasma membrane protusions [4]. Indeed, during neurogénesis, particles expressing CD133 have been shown in the neural tube lumina, where the release of CD133 expressing membrane particles seems to be a widespread event [6]. Furthermore, the human CD133 stem cell antigen is also expressed by different human epithelial cells including gut, and targeted to plasma membrane protusions [5].

CD133 is presently considered a useful marker to identify CSCs in colorectal cancer. It is of interest as to whether CD133 positive cells can be considered a prognostic factor. This study has immunohistochemically analyzed CD133 expression and assessed survival in a large series of stage II colon cancer and we found that CD133 expression was not an independent risk factor associated with recurrence or patient survival.

The relationship between CD133 expression and prognosis in colorectal carcinomas was examined previously. Horst *et al* reported that CD133 expression is an independently prognostic marker [7] whereas this kind of correlation was not observed by Kojima *et al* [8]. However some of these series were based on heterogeneous situations, involving rectal and colon cancer and different tumor stages (I–IV). Li *et al* analyzed a homogeneous IIIB group (104 patients) and showed that CD133 positive cancer cells contributed to the progression of colon cancer [9]. Nian-Hua Zang *et al* analyzed a group of stage II and III colon cancer founding a negative correlation survival in patients with co-expression con CDXCR4 and CD133 [10].

The discrepancy between the different studies might be derived from inadequate patient quantity and the mixed tumor stage (stage I to IV and colon and rectal cancer).

On the other hand, it is unlikely the CSCs can be characterized by only one marker. Stem cell-like properties have recently been reported in CD133 negative cells, as well. Therefore, the CD133 positive tumor cells may not represent the entire cancer-initiating population. A combination of cell surface markers is need for the definition of colon cancer stem cells. However, the cancer stem cell population is heterogeneous. Therefore, the levels of expression of the different cancer stem cell markers is variable, as a result of the biological function diversity of each of them.

NANOG, a homeodomain transcription factor, is an essential regulator for promotion of self-renewal of embryonic stem cells and inhibition of their differentiation. Previous studies indicated that NANOG is expressed in colon cancer cells, and suggested that their expression contributes to proliferation of colon cancer cells [11]. Moreover, several studies have shown that NANOG regulates different functions of cancer development, such as tumor cell proliferation, drug-resistence and cancer cell communication with the surrounding stroma, leading to cell motility, epithelial-mesenchymal transition and immune evasion [12].

We decided to analyze NANOG in this cohort of patients. NANOG and CD133 expression was observed in the same subset of neoplastic cells. However, NANOG levels of expression were higher than those of CD133, as determined by NANOG positive/CD133 negative neoplastic cells, that were shown as well. In addition, NANOG expression was also observed in stroma cells, probably related with the microenvironment communication function.

We found no correlation between CD133 or NANOG expression and survival (relapse free survival and overall survival).

Therefore, CD133 positive cells themselves may not have biological ability associated with malignant potential in colorectal cancer.

Previous studies using cell suspensions of colon cancer specimens demonstrated that CD133 positive cells were CK20 negative [3]. In our cohort 78% of samples were CK20 positive (defined by >10% positivity), and also no correlation was found with survival.

Several factors might explain the discrepancies with previous reports: a) mixed tumor stages where we analyze a very homogenous one, b) different antibodies used for the detection of the CD 133 molecule. Indeed, different results can be obtained by using different antibodies [13], [14] c) different criteria used to identify positive staining and different cut-off used to discriminate positive and negative tumors. In our study we have established four levels of positivity: <10%, 11–25%, 26–50% and >50%. Others considered positive if >50% positivity, (Horst *et al*), >10% (Kojima *et al*) or >5% (Li *et al*), d) inadequate patient cohort, this could explain the negative results for correlation in such a cohort with low recurrence expectancy, resulting only in a 17% of the cases. Nevertheless, we could demonstrate a correlation with known clinicopathologic factors and survival.

Additionally, we observed a high level of CD133 and NANOG positivity in this group of patient in a more initial stage, 60% CD133 positivity. Moreover, in 22% it was higher than 50%. We also found 94% NANOG positivity and 51% of them were higher than 50%. These results go against with those found by Li *et al,* in which up to 40% of cases in stage IIIB had <5% positivity.

This unexpected behavior of CD133 expression found in our study, higher in lower stage tumors than in more advanced lesions, has been observed in other reports [15]. This distribution is consistent with previous findings in a mouse model of colon carcinogenesis [16] and in human primary colon cancers [16]. In mouse colon carcinogenesis a significantly increased expression of CD133 have been observed, assessed by immunohistochemistry, in early neoplastic lesions which tended to decrease with tumor development [17] and an increased CD133 expression was reported in Dukes A compared to Dukes B and C colon cancers [16]. Previous studies reported that the intensity of CD133 is cell cycle-dependent and increased CD133 positive cells are correlated with increased DNA content, and all these findings are in agreement with the proposed ability of the protein to specifically identify tumor initiating cells which is related with the growth of both primary and metastatic disease [18] and thus mainly involved in the most active phases of tumor development.

This different CD133 expression through different stages has also been suggested by Shmelkov *et al*, who proposed that during metastasis, CD133 positive tumor cells generate CD133 negative cells, which are more aggressive and also able to initiate tumors in nude mice. The authors reported that 40% of metastatic tumors in their study were CD133 negative [19].

In **conclusion**, in this study of 100 stage II colon cancer patients, Stem Cell quantification defined by CD133 and NANOG expression has no correlation with RFS or OS. However, a correlation with established prognostic pathological factors was found. Therefore, our results suggest that CSCs may not play a major role in early phases of colon cancer.
